# Childhood modifiable risk factors and later life chronic kidney disease: a systematic review

**DOI:** 10.1186/s12882-023-03232-z

**Published:** 2023-06-22

**Authors:** Conghui Liu, Ye He, Alison J. Venn, Matthew D. Jose, Jing Tian

**Affiliations:** 1 Menzies Institute for Medical Research, University of Tasmania, 17 Liverpool Street, Hobart, TAS 7000 Australia; 2 The First Affiliated Hospital of Anhui Medical University, Hefei, Anhui China; 3 School of Medicine, University of Tasmania, Hobart, TAS Australia

**Keywords:** Childhood, Risk factors, Adulthood, Chronic kidney disease, Cohort studies

## Abstract

**Background:**

Relationships between adulthood modifiable risk factors and chronic kidney disease (CKD) are well-established, but associations with childhood risk factors are unclear. This study systematically assesses the published evidence about childhood modifiable risk factors and adulthood CKD.

**Methods:**

We searched MEDLINE, EMBASE, and Web of Science to 6^th^ May 2022. Articles were included if (1) they were population-based longitudinal studies, (2) exposures were potentially modifiable, for example through pharmacological or lifestyle modifications, including clinical conditions/measures (diabetes, blood pressure, adiposity, and dyslipidaemia); health behaviours (smoking, alcohol consumption, physical activity, fitness, and poor nutrition); and socio-economic factors (socio-economic position), and occurred during childhood (ages 2–19 years), and (3) outcome was CKD or surrogate markers of CKD in adulthood (ages 20 years or older). Three reviewers independently extracted the data.

**Results:**

15,232 articles were identified after deduplication; 17 articles met the inclusion criteria, reporting childhood blood pressure (n = 8), adiposity (n = 4), type 2 diabetes (n = 1), socio-economic position (n = 1), famine (n = 1), cardiorespiratory fitness (n = 1), and a healthy lifestyle score (n = 1). The results suggested positive associations of childhood adiposity, type 2 diabetes, and low socio-economic position and cardiorespiratory fitness in females with CKD in adulthood. Findings were inconsistent on associations between childhood BP and CKD in adulthood. Childhood healthy lifestyle score and exposure to famine were not associated with risk of CKD in adulthood.

**Conclusions:**

The limited evidence suggests childhood factors may contribute to the CKD risk in adulthood, particularly adiposity, type 2 diabetes, and low socio-economic position and cardiorespiratory fitness in females. Further high-quality community-based studies are needed with long-term follow-up and investigation of a broader range of modifiable risk factors.

**Supplementary Information:**

The online version contains supplementary material available at 10.1186/s12882-023-03232-z.

## Introduction

Chronic kidney disease (CKD) is a leading public health issue. From 1990 to 2016, overall global deaths due to CKD increased from 599,200 to 1,186,560, a rise of 98% [1]. CKD was ranked as the 12^th^ leading cause of death in 2016 and is estimated to become the 5^th^ by 2040 [2]. CKD typically has no symptoms at early stages and is often detected too late to delay the deterioration. Clinical guidelines in Australia recommend referral to a nephrologist once estimated glomerular filtration rate (eGFR) drops below 30 mL/min/1.73m^2^, yet a quarter present to nephrologists very late and commence dialysis within 90 days, missing the timely treatment opportunity to prevent the progression of the disease [3, 4]. Fortunately, CKD is largely preventable because kidney functional plasticity is substantial in infants, children, and even adults [5] and modifiable risk factors such as adiposity, insufficient physical activity (PA), and smoking are responsible for the major share of the CKD burden [6, 7]. Therefore, identification of childhood risk factors may help with early diagnoses and interventions, to prevent or delay the onset and progression of CKD in later life.

The relationships between modifiable risk factors in adulthood (e.g., obesity, smoking, hypertension, diabetes) and the onset of CKD are well-established [8–11], but associations with childhood risk factors are less clear. Recently, a prospective study involving 38,589 participants in the International Childhood Cardiovascular Cohorts Consortium demonstrated that childhood risk factors including body mass index (BMI), systolic blood pressure (SBP), total cholesterol level, triglyceride level, and youth smoking were associated with cardiovascular events in adulthood, but they did not investigate the relationships with kidney disease in adulthood [12]. A narrative review in 2017 identified that early-life adverse events could cause structural and functional changes in the development of the kidney, whereby individuals exposed to early-life risk factors such as maternal malnutrition, preterm birth, and some medications after birth may be vulnerable to developing CKD in later life [13]. Another narrative review also suggested that the risk of CKD can be increased by multiple factors present in childhood including genetic factors, perinatal factors (e.g., prematurity), childhood disease, and lifestyle factors [14]. However, the absence of objective and systematic selection criteria may have led to bias in the selection of papers and thereby makes the interpretation of results more difficult [15]. A comprehensive and systematic review is needed to better identify whether potentially modifiable risk factors in childhood, including clinical conditions/measures (diabetes, BP, adiposity, and dyslipidaemia); health behaviours (smoking, alcohol consumption, PA, fitness, and poor nutrition); and socio-economic factors (SEP) predict incident CKD in adulthood.

To fill this research gap, we aimed to perform a systematic review of the existing literature on associations between childhood modifiable risk factors and CKD in adulthood including surrogate markers of CKD.

## Methods

This systematic review was reported in accordance with the Meta-analysis of Observational Studies in Epidemiology (MOOSE) [16].

A systematic hand literature search was performed in MEDLINE, EMBASE, and Web of Science databases for articles published prior to 4^th^ March 2021. The update was conducted to May 6, 2022. The search strategy was implemented by the research team. A research librarian assisted in planning the search and helped to create correct search strings. No language restriction was enforced. The detailed search terms in each database are in Appendix 1.

Eligibility criteria for included studies were as follows: (1) the study was a population-based longitudinal study; (2) the exposures of interest were measured in childhood (ages 2–19 years) and included potentially modifiable risk factors (those can be changed or controlled with pharmacological or lifestyle interventions): clinical conditions/measures (diabetes, BP, adiposity, and dyslipidaemia); health behaviours (smoking, alcohol consumption, PA, fitness, and poor nutrition); and SEP; (3) the outcomes of interest were diagnosed or evaluated in adulthood (ages 20 years or older), and included dichotomous outcomes of the onset of CKD (as defined in each paper), presence of kidney damage (e.g. albuminuria) or decreased kidney function (eGFR); or continuous outcome values of urinary albumin-creatine ratio (UACR) and/or eGFR. Reviews, non-human studies, and non-modifiable childhood risk factors including genetic causes, and congenital anomalies of kidney and urinary tract (CAKUT) were excluded.

The initial screening was performed by one reviewer (CL) by assessing the titles, abstracts and keywords and was set to be relatively broad to retain as many relevant studies as possible. Two reviewers (CL and YH) then independently screened full-text articles identified from the initial screening to ensure all inclusion criteria were met. 600 records were randomly selected for the third reviewer (JT) to check the consistency. The Cohen’s kappa coefficient was 1.00. An Inclusion/Exclusion form was used to collect useful information from included studies (Appendix 2). Reference lists and the bibliographies of included studies and review articles were also scrutinized to identify any further original articles. Discrepancies were resolved by discussion or by including a third reviewer (JT) to reach a consensus. We did not search for unpublished articles. Endnote X20 (http://www.endnote.com) was used to manage the located records.

Methodological quality of included studies was independently assessed by three reviewers (CL, YH, and JT) using the adapted Newcastle-Ottawa Quality Assessment Scale (NOQAS) [17] (Appendix 3). Assessment of methodological quality involved three aspects: participants selection and exposure measurement, cohort comparability based on study design or analysis, and outcomes assessment and follow-up adequacy. Studies were scored by three authors with grades from 0 to 9, with 0–3 as poor quality, 4–6 as fair quality, 7–9 as high quality. When necessary, a fourth reviewer (AV) was involved in a discussion to reach a consensus.

## Results

**Fig. 1:**
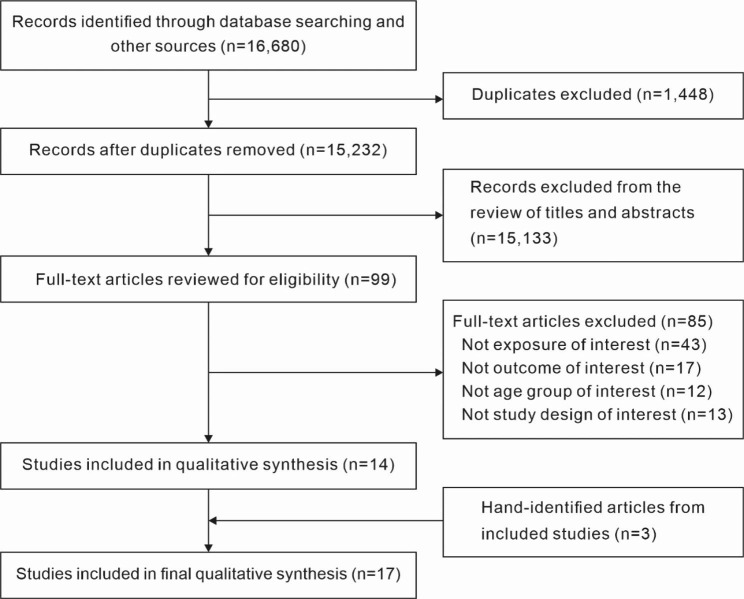
Flow chart of articles identified in search and included in the systematic review

A total of 16,680 articles were retrieved from an initial search in three electronic databases. After deduplication (n = 1,448), the titles, abstracts, and keywords of 15,232 records were initially reviewed, with 99 potentially relevant articles undergoing full-text review. Of these articles, 85 were excluded for various reasons including ineligibility of the exposure or outcome (n = 60), the age group (n = 12), or the study design (n = 13), leaving 14 included articles. Three articles were further identified from a hand-search of included studies; therefore, 17 articles were kept in this systematic review (Fig. 1).

Characteristics of the 17 eligible studies are summarized in Table 1. The follow-up length ranged from 8.5 to 62 years. The earliest study was published in 2002, and the latest in 2022. The sample size ranged from 412 to 6,267. About half of the studies examined the exposure of BP (n = 8) [18–25], while the rest examined adiposity (n = 4) [26–29], type 2 diabetes (T2D, n = 1) [30], SEP (n = 1) [31], famine (n = 1) [32], cardiorespiratory fitness (CRF, n = 1) [33], and a healthy lifestyle score (HLS) which was generated from the sum scores of five lifestyle factors (BMI, smoking, alcohol consumption, PA, and diet) (n = 1) [34]. The 17 articles were from five countries [China (n = 9) [19, 21, 22, 24, 25, 27, 29, 32, 35], USA (n = 3) [18, 20, 30], Australia (n = 3) [28, 33, 34], UK (n = 1) [26], and Ireland (n = 1) [31]], and eight cohorts [Hanzhong Adolescent Hypertension Cohort (n = 6) [22, 24, 25, 27, 29, 35], Childhood Determinants of Adult Health (CDAH) study (n = 3) [28, 33, 34], Bogalusa Heart Study (n = 2) [18, 20], Beijing BP Cohort (n = 2) [19, 21], Gila River Indian Community Study (n = 1) [30], 1946 British Birth Cohort (n = 1) [26], Irish Longitudinal Study (n = 1) [31], and the China Health and Retirement Longitudinal Study (n = 1) [32]]. All studies included both males and females. For the outcomes, ten studies reported the onset of CKD or subclinical kidney damage (SKD) [22, 24–28, 31, 32, 34, 35]; ten studies reported the onset of albuminuria or the level of UACR [18, 19, 21, 22, 25, 27–30, 33]; five studies reported the level of eGFR [20–22, 27, 28]; one reported the onset of glomerular hyperfiltration (GHF) [33].

**Table 1:** Listing of articles about childhood modifiable risk factors for later life chronic kidney disease ^a^

ID	Author	Year	Country	Study name	Target population	Sample size	Baseline age (years)	Mean FU length (years)	Exposure in childhood	Outcome(s) in adulthood	Main finding(s)
1	Hoq et al. [18]	2002	USA	Bogalusa Heart Study	School-aged children and young adults in a biracial population	2,122	5–17	16	BP, annual change in BP from childhood to adulthood	Microalbuminuria ^b^	Childhood BP and annual change in BP from childhood to adulthood were associated with microalbuminuria in Blacks, but not in Whites.
2	Zhao et al. [19]	2008	China	Beijing BP Cohort	Primary and secondary school-aged children	412	6–18	18	Change in HTN status from childhood to adulthood	Microalbuminuria ^b^	High BP only in childhood was not associated with microalbuminuria in adults ^c^.
3	Yan et al. [20]	2018	USA	Bogalusa Heart Study	School-aged children and young adults in a biracial population	2,512	4–19	25	BP, BP from childhood to adulthood	eGFR	Childhood BP was not associated with adult eGFR in Blacks and Whites. Long-term burden of BP, reflected by total BP area under the curve value, was significantly and negatively associated with adult eGFR in Blacks.
4	Yan et al. [21]	2018	China	Beijing BP Cohort	Children from primary and secondary schools	1,222	6–18	23	BP, change in HTN status from childhood to adulthood	Microalbuminuria ^b^; eGFR	High BP in childhood was not associated with microalbuminuria, and eGFR in adulthood.
5	Zheng et al. [22]	2018	China	Hanzhong Adolescent Hypertension Cohort	School-aged children from rural areas	2,430	6–15	30	BP trajectories from childhood to adulthood	UACR; eGFR; SRD ^d^	Compared to the low stable trajectory group, higher BP trajectories were associated with a higher level of UACR and a higher risk of SRD in adulthood, but not with eGFR in adulthood ^e^.
6	Liao et al. [35]	2020	China	Hanzhong Adolescent Hypertension Cohort	School-aged children from rural areas	1,738	6–15	30	Elevated BP; pre-HTN; HTN ^f^	SRD ^d^	Elevated BP and prehypertension in childhood was not associated with SRD in adulthood. Hypertension in childhood was associated with the risk of SRD in adulthood.
7	Liao et al. [24]	2021	China	Hanzhong Adolescent Hypertension Cohort	School-aged children from rural areas	1,738	6–15	30	Pulsatile stress; change in pulsatile stress from childhood to adulthood	SRD ^d^	Pulsatile stress in childhood was associated with adult SRD, especially in males. High pulsatile stress in childhood but normal in adulthood still had an increased risk of SRD in males.
8	Wang *et a*l. [25]	2022	China	Hanzhong Adolescent Hypertension Cohort	School-aged children from rural areas	1,771	6–18	30	BPV	SKD ^d^; albuminuria ^g^	BPV from childhood to middle adulthood was associated with higher risk of SKD and albuminuria in adulthood.
9	Kim et al. [30]	2010	USA	Gila River Indian Community Study	Members of the Gila River Indian Community	2,666	5–19	8.1 ^h^	type 2 diabetes	Macroalbuminuria ^i^	The incidence of macroalbuminuria in adulthood was higher in diabetic children than nondiabetic children.
10	Silverwood et al. [26]	2013	UK	1946 British Birth Cohort	Socially stratified sample of singletons in England, Scotland, and Wales	4,340	2–20 ^j^	62	Overweight latent classes between ages 2 and 20 years	CKD ^k^	Being overweight in early years was associated with a high risk of CKD in later life.
11	Yan et al. [27]	2021	China	Hanzhong Adolescent Hypertension Cohort	School-aged children from rural sites of towns	2,162	6–15	30	BMI trajectories	SRD ^d^, eGFR, UACR	Child-to-adult BMI trajectories that worsen or persist at high levels were associated with an increased risk of SRD, but not with levels of eGFR and UACR ^e^.
12	Liu et al. [28]	2021	Australia	Childhood Determinants of Adult Health study	A nationally representa-tive sample of school children	1,442	7–15	33	BMI trajectories	SKD ^d^, eGFR, UACR	Higher BMI trajectories were associated with a higher risk of SKD in midlife. The relationship with eGFR was inconsistent. No significant association was found with UACR.
13	Wang et al. [29]	2022	China	Hanzhong Adolescent Hypertension Cohort	School-aged children from rural sites of towns	4,623	6–18	30	BMI trajectories	Albuminuria ^g^	Higher BMI trajectories were associated with an increased risk of albuminuria in midlife.
14	Canney et al. [31]	2018	Ireland	The Irish Longitudinal Study on Aging	Cluster-sampled community-dwelling adults aged ≥ 50 years	4,996	≤ 14 ^l^	48 ^l^	SEP measured by father’s occupation	CKD	Low childhood SEP was associated with an increased risk of CKD in women, independent of adulthood SEP. Similar association was absent in men.
15	Lv et al. [32]	2020	China	China Health and Retirement Longitudinal Study	Middle-aged and elderly population in mainland China	6,267	Preschool; school-aged ^k^	52 ^m^	Famine	CKD	Preschool and school-aged exposure to famine was not associated with the risk of CKD.
16	Liu et al. [33]	2022	Australia	Childhood Determinants of Adult Health study	A nationally representative sample of school children	1,371	7–15	33	CRF ^n^	GHF ^o^, albuminuria ^g^	Childhood low CRF was associated with an increased risk of GHF in women, but not in men. No significant association was found with albuminuria.
17	Liu et al. [34]	2022	Australia	Childhood Determinants of Adult Health study	A nationally representative sample of school children	750	7–15	33	HLS ^p^	SKD ^d^	Childhood HLS was not associated with the risk of SKD in adulthood.

FU, follow-up; BP, blood pressure; HTN, hypertension; CKD, chronic kidney disease; SKD, subclinical kidney damage; SRD, subclinical renal damage; eGFR, estimated glomerular filtration rate; UACR, urinary albumin-to-creatinine ratio; BPV, blood pressure variability; SEP, social economic position; CRF, cardiorespiratory fitness; GHF, glomerular hyperfiltration; HLS, healthy lifestyle score.

^a^ All studies used the design of a prospective cohort study except ID14 and ID15 (retrospective cohort study). All outcomes were measured objectively. All studies included both males and females.

^b^ Microalbuminuria defined as a UACR between 3 and 30 mg/mmol.

^c^ Relationship was analysed by chi-square test.

^d^ SKD and SRD defined as eGFR between 30 and 60 ml/min per 1.73 m^2^ or UACR of at least 2.5 mg/mmol in men and 3.5 mg/ mmol in women.

^e^ The relationship with UACR and eGFR was analysed using Kruskal–Wallis test and one-way ANOVA.

^f^ Elevated BP was defined as SBP/DBP ≥ 90th percentile by BPRS (Blood Pressure Reference Standard Tables of Chinese children aged 3–17 years old) tables or > 120/80 mm/Hg; prehypertension was defined as SBP/DBP ≥ 90th percentile and < 95th percentile with the use of the BPRS tables or SBP/DBP > 120/80 mmHg); hypertension was defined as SBP/DBP ≥ 95th percentile with the use of the BPRS tables.

^g^ Albuminuria was defined as UACR ≥ 30 mg/g.

^h^ Median.

^i^ Macroalbuminuria defined as UACR ≥ 300 mg/g.

^j^ Height and weight were measured at ages 2, 4, 6, 7, 11, and 15 and self-reported at age 20 years.

^k^ CKD defined as eGFR < 60 mL/min/1.73 m^2^ or UACR ≥ 3.5 mg/mmol.

^l^ Participants aged 55–69 years old retrospectively reported their father’s occupation when they were growing up, until the age of 14 years.

^m^ Birth year was used as the proxy variable for famine exposure in 1959–1962, when famine status peaked. Famine exposure was then divided into fetal exposed, preschool exposed, and school-aged exposed. Outcome was measured at age of 44 to 61 years.

^n^ CRF in 7–15 years old was estimated by age and sex-specific time taken to complete a 1.6 km (1 mile) run.

^o^ GHF was defined as the upper 5th percentile value of eGFR, standardized for age and sex.

^p^ The HLS was generated from the sum scores of five lifestyle factors (body mass index, smoking, alcohol consumption, physical activity, and diet). Each factor was scored as poor (0 point), intermediate (1 point), or ideal (2 points).

Table 2 summarizes the results of the methodological quality assessment using NOQAS for each included study. The quality score ranged from 6 to 8.5 out of 9. Most studies were scored as high quality (n = 15), only a few studies had fair quality (n = 2), and no study had poor quality. All studies had somewhat representative samples of the population of interest. Most studies collected exposure data from objective measurement or structured interview (n = 15) but did not verify that the outcome of interest (e.g., albuminuria) was not present at the start of the study in childhood (n = 11). The other two studies did not objectively measure the exposure due to the characteristics of the studied exposure: SEP [31] and famine [32]. All studies controlled for age and sex during study design or analysis. For the assessment of outcomes, all studies collected data from objective measurement. One study had a follow-up rate of more than 80% [26], and nine studies compared the study sample with those lost to follow-up [21, 22, 25, 27, 28, 31, 33–35].

**Table 2:** Summary of quality assessment

					Selection				Comparability		Outcome		
ID	Author	Year	Total scores	Grade ^a^	Represen-tativeness. of the exposed cohort	Represen-tativeness. of the non-exposed cohort	Ascertainment of the exposure	Outcome of interest was not present at start of study	Controls for age and sex	Considers other relevant covariates	Outcome assessment	Adequacy of follow-up length ^b^	Adequacy of follow-up of cohorts
1	Hoq et al. [18]	2002	7	High	*	*	*	0	*	*	*	*	0
2	Zhao et al. [19]	2008	7	High	*	*	*	0	*	*	*	*	0
3	Yan et al. [20]	2018	7	High	*	*	*	0	*	*	*	*	0
4	Yan et al. [21]	2018	7.5	High	*	*	*	0	*	*	*	*	0.5*
5	Zheng et al. [22]	2018	8.5	High	*	*	*	*	*	*	*	*	0.5*
6	Liao et al. [35]	2020	8.5	High	*	*	*	*	*	*	*	*	0.5*
7	Liao et al. [24]	2021	8.5	High	*	*	*	*	*	*	*	*	0.5*
8	Wang et al. [25]	2022	8.5	High	*	*	*	*	*	*	*	*	0.5*
9	Kim et al. [30]	2010	7	High	*	*	*	0	*	*	*	*	0
10	Silverwood et al. [26]	2013	8	High	*	*	*	0	*	*	*	*	*
11	Yan et al. [27]	2021	7.5	High	*	*	*	0	*	*	*	*	0.5*
12	Liu et al. [28]	2021	7.5	High	*	*	*	0	*	*	*	*	0.5*
13	Wang et al. [29]	2022	8.5	High	*	*	*	*	*	*	*	*	0.5*
14	Canney et al. [31]	2018	6.5	Fair	*	*	0	0	*	*	*	*	0.5*
15	Lv et al. [32]	2020	6	Fair	*	*	0	0	*	*	*	*	0
16	Liu et al. [33]	2022	7.5	High	*	*	*	0	*	*	*	*	0.5*
17	Liu et al. [34]	2022	7.5	High	*	*	*	0	*	*	*	*	0.5*

^a^ Studies were scored from 0 to 9, with 0–3 as poor quality, 4–6 as fair quality, 7–9 as high quality.

^b^ Whether follow-up is long enough for outcomes to occur.

### Blood pressure

We identified eight studies that examined the association between childhood BP and CKD in adulthood. Two of them used data from the Bogalusa Heart Study, USA [18, 20], and the remainder were from China: two from the Beijing BP Cohort [19, 21] and four from the Hanzhong Adolescent Hypertension Cohort [22, 24, 25, 35]. Two studies analysed childhood BP exclusively as a continuous variable [18, 20], one exclusively as a dichotomous variable [35], and two as both continuous and dichotomous variables [19, 21]. One study examined the BP trajectory from childhood to adulthood [22]; one study examined pulsatile stress (resting heart rate × pulse pressure [PP = SBP minus diastolic BP]) in childhood [24]; and one study examined BP variability (BPV) from childhood to adulthood [25].

The association between childhood BP and the risk of microalbuminuria or SKD in adulthood was examined in four articles [18, 19, 21, 35]. Two of the four articles did not find significant associations [19, 21]; one of the four articles stratified analyses by race and revealed a positive relationship of childhood BP and the annual change in BP from childhood to adulthood with microalbuminuria in adulthood in Blacks, but not in Whites [18]; the remaining article found that childhood hypertension (SBP/ diastolic [DBP] ≥ 95th percentile of Chinese reference standards for children aged 3–17 years old) was associated with a higher risk of SKD in adulthood, but this association was absent in children with prehypertension (SBP/DBP ≥ 90th percentile and < 95th percentile) [35]. Of note, one of the four articles used chi-square test for analyses, with no consideration of covariates [19]. The association between childhood BP and eGFR in adulthood was examined in two articles with neither detecting a significant association [20, 21]. One article examined the relationship of BP trajectory from childhood to adulthood with the risk of SKD and continuous levels of UACR and eGFR in adulthood [22]. It found higher SBP trajectories were associated with an increased risk of SKD and higher levels of UACR in adulthood, but not with eGFR levels. Similar results were reported for DBP and mean arterial pressure trajectories [22]. Of note, the analyses for UACR and eGFR in this study were performed using Kruskal–Wallis tests and one-way ANOVA respectively [22], and these statistical tests cannot take confounders into account. Pulsatile stress in childhood was examined and it was found that high pulsatile stress (above the highest quartile) in childhood was associated with an increased risk of SKD in male adults [24]. In addition, higher BPV from childhood to adulthood was significantly associated with higher risks of SKD and albuminuria in adulthood [25].

### Type 2 diabetes

Only one study reported the relationship of childhood T2D with adulthood CKD [30]. It found the incidence of macroalbuminuria was higher in participants with T2D in childhood (ages 5 to 19 years) than those without T2D (incidence rate ratio = 15.9, 95% confidence interval [CI], 11.1–22.6).

### Adiposity

We identified four studies examining how body weight trajectories from childhood to adulthood may affect adulthood CKD [26–29]. Although all the studies used BMI as an adiposity indicator, one of them analysed BMI categorically [26] according to international overweight cut-offs by Cole, et al. [36], and the other three analysed BMI as a continuous variable [27–29]. Utilising at least one from seven repeated measurements of weight status from ages 2 to 20 years (2, 4, 6, 7, 11, 15, and 20 years), the 1946 British Birth Cohort study modelled the early-life overweight latent classes and found that being overweight in early life was associated with an increased risk of CKD in later life (ages 60 to 64 years) [26]. The odds ratio (OR) of CKD for participants in the pubertal-onset- or always-overweight latent classes was 2.04 (95%CI, 1.09–3.82) compared with those in the never-overweight latent class [26]. Two studies from the Hanzhong Adolescent Hypertension Cohort modelled the trajectories of continuous BMI from ages 6 to 45 years [27, 29]. One of the two studies identified four BMI trajectories (stable normal, moderately increasing overweight, resolving, and progressively increasing obese) using latent class growth mixture modelling (LCGMM) and found that BMI trajectories that worsen or persist at high levels from childhood to adulthood were associated with an increased risk for SKD, but not related to high levels of UACR and eGFR in adulthood (ages 36 to 45 years) [27]. Furthermore, individuals in the resolving group (BMI from high to low) exhibited a similar risk of SKD to those in the stable normal group [relative risk (RR) = 1.14, 95%CI, 0.75–1.74]. Notably, the comparisons of UACR and eGFR by different BMI trajectories were performed using Kruskal-Wallis test and one-way ANOVA only [27], without consideration of the effects of confounders on the results. The other study identified three BMI trajectories (low-increasing, moderate increasing, and high increasing) using group-based trajectory modelling and found that higher BMI trajectories were associated with higher levels of UACR and an increased risk of albuminuria in adulthood, especially in males [29]. Similar findings were observed in the CDAH study where participants with increasing BMI trajectories from childhood to adulthood had an increased risk of SKD in midlife [28].

### Socioeconomic position

One study was identified examining associations of childhood SEP with CKD in adulthood [31]. Using retrospectively reported father’s occupation to reflect the childhood SEP, this study showed that childhood SEP was negatively associated with later-life CKD in women (OR = 2.09, 95%CI, 1.38–3.18), which was independent of occupation in adulthood (OR = 1.90, 95%CI, 1.24–2.92), while no significant association was found in men. Furthermore, compared with women who had persistently high SEP from childhood to adulthood, those with an upward SEP trajectory (low to intermediate/high, and intermediate to high) still had an increased odds of CKD in adulthood (OR = 1.85, 95%CI, 1.05–3.25) [31].

### Nutrition

Only one study relating to nutrition was identified evaluating the influence of childhood exposure to famine on CKD outcomes in adulthood [32]. In this study, the odds of CKD among individuals with fetal, preschool, and school-aged exposure to a Chinese famine (from 1959 to 1962) was significantly higher than for those without famine exposure (OR = 1.79, 95%CI, 1.14–2.80; OR = 2.28, 95%CI, 1.50–3.45; OR = 4.25, 95%CI, 2.86–6.32, respectively). However, the statistical significance disappeared after adjusting for confounders (OR = 1.03, 95%CI, 0.54–1.97; OR = 0.73, 95%CI, 0.25–2.10; OR = 0.89; 95%CI, 0.21–3.72, respectively). Further stratified analyses by sex and famine severity found that only men with fetal exposure to severe famine had higher odds of CKD before (OR = 2.63, 95%CI,1.21–5.70) and after adjustment for confounders (OR = 2.05, 95%CI, 0.74–5.65) compared with non-exposed men.

### Cardiorespiratory fitness

One study was identified investigating the relationship of childhood CRF (estimated by age and sex-specific time taken to complete a 1.6-km run) with GHF and albuminuria in adulthood [33]. In this study, lower CRF in childhood was associated with an increased risk of GHF in adulthood for females. Compared with individuals with high childhood CRF, the average RR was 2.86 (95%CI, 1.04–7.86) for individuals with moderate childhood CRF, and was 3.38 (95%CI, 1.13–10.14) for individuals with low childhood CRF. Whereas no significant associations were found with GHF in males or albuminuria in either males or females.

### Healthy lifestyle score

We found one study investigating clustering of five lifestyle factors (BMI, smoking, alcohol consumption, PA, and diet) to generate a HLS and its association with the risk of SKD in adulthood [34]. No significant associations were found between HLS in childhood, or from childhood to adulthood, and the risk of SKD in adulthood.

## Discussion

To our knowledge, this is the first systematic review synthesizing the published evidence on the longitudinal associations of childhood modifiable risk factors with CKD in adulthood. Seventeen studies based on eight cohorts were identified as eligible to be included and we had four key findings on the relationship with CKD or surrogate markers of CKD in adulthood:

1. Inconsistent associations were found with childhood BP, though positive associations were found with high BP and BMI trajectories from childhood to adulthood.
2. Some associations were found with childhood T2D, low SEP (in females), low CRF (in females), and poor nutrition (famine), but current evidence is very limited.
3. No associations were found with a HLS of five lifestyle factors (BMI, smoking, alcohol consumption, PA, and diet) in childhood, or from childhood to adulthood.
4. No study has reported the individual relationships with childhood smoking, alcohol consumption, PA, or cholesterol.

Taken together, previous population-based longitudinal studies suggested that modifiable risk factors for the onset and development of CKD can appear as early as childhood, but existing evidence is limited and inconsistent.

High BP is one of the most important modifiable risk factors contributing to the development of CKD. Previous studies have reported that high BP in adulthood, and increasing BP over time in adulthood, were associated with an increased risk of CKD [37, 38]. Given the tracking of high BP from childhood to adulthood, it would be expected that those with high BP in childhood might have a higher risk of CKD than their peers without high BP [39]. However, in this review, we found the evidence was inconsistent. A possible explanation is the low statistical power in studies with a low prevalence of CKD, particularly in younger study populations when CKD is expected to be at an early stage [40, 41]. A significant relationship was more likely to be found in an older population with worse kidney function and a higher prevalence of CKD. However, four out of eight BP related studies did not verify that CKD was not present at the start of the study when childhood exposures were measured, so it was not possible to discount that children with a high BP already had decreased kidney function.

A previous systematic review and meta-analysis of 39 cohorts covering 630,677 adults suggested that obesity in adulthood was associated with a higher risk of low eGFR, albuminuria, and CKD [7]. In this systematic review, we identified four studies that included childhood adiposity in trajectories of BMI and overweight status respectively, and each found significant associations with adulthood CKD. To the best of the authors’ knowledge, no study has examined whether childhood adiposity is an independent risk factor for adult CKD irrespective of their subsequent body weight status. Nevertheless, the finding that participants who had the greatest BMI increase or highest BMI trajectories from childhood to adulthood had the highest risk of CKD indicates that the effects of high BMI on later life CKD may be accumulated [26–29].

The importance of using a life-course approach to prevent or delay the onset and development of CKD by controlling risk factors from childhood to adulthood has been emphasized by many international organizations including the World Health Organization, the World Report on Aging and Health, and Children’s and Adolescents’ Health [42]. Various life-course models that reflect the pathways from early to later life diseases have been proposed including the accumulation of risk model, chain of risk model, the critical period model, and the sensitive period model [43]. In this review, only one study of childhood SEP and CKD in adulthood tested different hypotheses relating to life-course models and found that women whose SEP decreased from childhood to adulthood had similar odds of CKD compared with those with persistently high SEP [31]. Future studies are needed to investigate the relationship of modifiable risk factors over the life-course from childhood with later life CKD by considering all possible life-course models in one study sample. Future studies might also investigate social factors such as racism and adverse childhood experiences which have been shown to be associated with poorer health [44, 45].

There are several gaps in prior studies that need to be pointed out. First, only 17 articles met the inclusion criteria of this systematic review; six of the 17 articles used data from the Hanzhong Adolescent Hypertension Cohort [22, 24, 25, 27, 29, 35], three were from CDAH study [28, 33, 34], two were from the Bogalusa Heart Study [18, 20], and another two were from the Beijing BP Cohort [19, 21]. Results from one cohort may only be applicable to a specific population and their interpretation should be made with caution. For example, participants included in the Hanzhong Adolescent Hypertension Cohort were from rural areas in northern China (98.2% were Han Chinese), and results might not be generalisable to other races or populations with high SEP. Second, most of the identified studies (n = 14) examined surrogate markers of CKD instead of CKD itself, which would be expected to have low prevalence in middle-aged individuals. Although ongoing follow-up of prospective cohort studies may capture the natural history of CKD development, loss to follow-up is hard to avoid along with reduced sample size and possible bias from loss of representativeness [46]. Furthermore, only five of the 17 studies verified that there was no chronic disease in the child’s medical history (all from Hanzhong Adolescent Hypertension Cohort), hence it is unclear whether kidney disease might have preceded exposure to the risk factors investigated. However, the prevalence of CKD in childhood is very low [47], thus the possible influence should be limited. Finally, chronicity (reduced kidney function for at least 3 months) of CKD was not available for all the included studies, which is a recommended criterion to define CKD [48]. This may lead to nondifferential misclassification (CKD misclassified equally between the study groups) and will generally bias the results toward the null.

This systematic review identified limitations in the literature, and publication bias may have influenced the findings, for example, if studies with negative findings were less likely to be published. We were unable to do a meta-analysis because the eligible articles were scarce, and the definitions of exposures were not consistent. For example, the exposure of BP included continuous BP, categorical BP, annual change in BP, BP trajectories, pulsatile stress, and BPV. This leads to heterogeneity in the analysis and limits clear recommendations in terms of possible interventions to combat CKD.

Although current evidence supporting the relationship between childhood modifiable risk factors and adult CKD is limited, the implications of these findings are in line with public health recommendations for preventing CKD and other chronic diseases such as cardiovascular disease [5], and a life-course approach to interventions across the life-course. Our findings suggest that interventions to reduce the burden of CKD in later life might include screening and management of high BMI, serum levels of glucose, and BP earlier in life from childhood to adulthood, especially in high-risk populations. However, the potential clinical implications of our findings should be interpreted cautiously since they are based on relatively few studies.

## Conclusions

The limited available evidence suggests that for some individuals, CKD may have its origins in childhood and approaches to prevention may need to start early in life. Further high-quality community-based longitudinal studies are needed to better understand the role of a wide range of modifiable risk factors in predicting CKD in adulthood and their potential as targets for CKD prevention.

## Electronic supplementary material

Below is the link to the electronic supplementary material.

Supplementary Material 1

## Data Availability

All data generated or analysed during this study are included in this published article.

## Abbreviations

BMI: body mass index
BPV: blood pressure variability
CAKUT: congenital anomalies of kidney and urinary tract
CDAH: Childhood Determinants of Adult Health
DBP: diastolic blood pressure
eGFR: estimated glomerular filtration rate
GHF: glomerular hyperfiltration
LCGMM: latent class growth mixture modelling
MOOSE: Meta-analysis of Observational Studies in Epidemiology
OR: odds ratio
PA: physical activity
PP: pulse pressure
RR: relative risk
SBP: systolic blood pressure
SEP: socio-economic position
SKD: subclinical kidney damage
T2D: type 2 diabetes
UACR: urinary albumin-creatine ratio
