# Supplementary material for: Childhood modifiable risk factors and later life chronic kidney disease: a systematic review
Source: BMC Nephrol. 2023 Jun 22;24:184. doi: 10.1186/s12882-023-03232-z (PMC10288726; doi:10.1186/s12882-023-03232-z)
Supplement: Supplementary file 1 — Supplementary Material 1 [file 12882_2023_3232_MOESM1_ESM.docx]

**Appendix 1. Search strategy**

A search strategy for MEDLINE

1 ((adolescen$2 or p?ediatric$1 or teenage$2 or child$4 or kid$1 or boy$1 or girl$1 or schoolchild$4 or schoolage$ or school age$ or youngster$1 or juvenile$) adj5 (socioeconomic status or socio-economic status or SES or socioeconomic position$1 or socio-economic position$1 or SEP or family income or parental income or parental education or parental occupation or smok$3 or tobacco or cigarette$1 or alcohol intake or alcohol drink$ or drink$ alcohol or wine$1 or liquor$1 or spirit$1 or physical activit$ or PA or aerobic exercise$1 or aerobic sport$1 or body mass index or BMI or abdominal obesity or overweight or over weight or over-weight or obesity or adiposity or high blood pressure or hypertension or diabetes or T2D or T1D or Glycated Hemoglobin A or Hemoglobin A1c or HbA1c or high blood glucose or low-density lipoprotein or LDL or high-density lipoprotein or HDL or very low density lipoprotein or VLDL or metabolic syndrome or MetS or Met or dyndrome X or triglycerides or triacylglycerol or triacylglyceride or TAG or TG or diet$ or nutrition$1 or nutritional deficienc$3 or dietary deficienc$3 or dystrophy)).ab,kf,ti.

2 exp Child/

3 exp Adolescent/

4 exp Pediatrics/

5 or/2-4

6 exp Socioeconomic Factors/

7 exp Income/

8 exp Education/

9 exp Occupations/

10 exp social support/

11 exp Smoke/

12 exp Smoking/

13 exp "Tobacco Use"/

14 exp Nicotine/

15 exp Alcohol Drinking/

16 exp Alcoholism/

17 exp Exercise/

18 exp Sports/

19 exp Physical Fitness/

20 exp body mass index/

21 exp Obesity/

22 exp Body Weight/

23 exp Obesity, Abdominal/

24 exp Pediatric Obesity/

25 exp Blood Pressure/

26 exp Hypertension/

27 exp Diabetes Mellitus/

28 exp Insulin/

29 exp Glycated Hemoglobin A/

30 exp Blood Glucose/

31 exp Lipids/

32 exp Dyslipidemias/

33 exp Hyperlipidemias/

34 exp Cholesterol/

35 exp cholesterol, hdl/ or exp cholesterol, ldl/ or exp cholesterol, vldl/

36 exp Lipoproteins/

37 exp Triglycerides/

38 exp Metabolic Syndrome/

39 exp Diet/

40 exp Nutrients/

41 exp Malnutrition/

42 exp Nutrition Disorders/

43 or/6-42

44 5 and 43

45 1 or 44

46 ((adult$1 or adulthood or middle aged or young adult$4 or older or old people or senior$1 or elderly) adj5 (chronic renal insufficiency or renal damage or kidney damage or kidney function or chronic kidney disease or CKD or chronic kidney failure or chronic kidney insufficiency or chronic kidney dysfunction or chronic renal failure or chronic renal dysfunction)).ab,kf,ti.

47 exp Adult/

48 exp Kidney Diseases/

49 exp Renal Insufficiency, Chronic/

50 exp Proteinuria/

51 exp Kidney Failure, Chronic/

52 exp Glomerular Filtration Rate/

53 or/48-52

54 47 and 53

55 46 or 54

56 exp Cohort Studies/

57 exp Case-Control Studies/

58 longitudinal.ab,kf,ti.

59 cohort stud$3.ab,kf,ti.

60 cohort analy$3.ab,kf,ti.

61 (follow up$1 or followup$1).ab,kf,ti.

62 case control stud$3.ab,kf,ti.

63 retrospective.ab,kf,ti.

64 prospective.ab,kf,ti.

65 or/56-64

66 45 and 55 and 65

A search strategy for EMBASE

1 ((adolescen$2 or p?ediatric$1 or teenage$2 or child$4 or kid$1 or boy$1 or girl$1 or schoolchild$4 or schoolage$ or school age$ or youngster$1 or juvenile$) adj5 (socioeconomic status or socio-economic status or SES or socioeconomic position$1 or socio-economic position$1 or SEP or family income or parental income or parental education or parental occupation or smok$3 or tobacco or cigarette$1 or alcohol intake or alcohol drink$ or drink$ alcohol or wine$1 or liquor$1 or spirit$1 or physical activit$ or PA or aerobic exercise$1 or aerobic sport$1 or body mass index or BMI or abdominal obesity or overweight or over weight or over-weight or obesity or adiposity or high blood pressure or hypertension or diabetes or T2D or T1D or Glycated Hemoglobin A or Hemoglobin A1c or HbA1c or high blood glucose or low-density lipoprotein or LDL or high-density lipoprotein or HDL or very low density lipoprotein or VLDL or metabolic syndrome or MetS or Met or dyndrome X or triglycerides or triacylglycerol or triacylglyceride or TAG or TG or diet$ or nutrition$1 or nutritional deficienc$3 or dietary deficienc$3 or dystrophy)).ab,kw,ti.

2 exp child/

3 exp adolescent/

4 exp pediatrics/

5 exp adolescent/

6 or/2-5

7 exp social status/

8 exp family income/

9 exp education/

10 exp occupation/

11 exp social support/

12 exp social class/

13 exp smoke/

14 exp smoking/

15 exp cigarette smoke/

16 exp tobacco/

17 exp tobacco consumption/

18 exp nicotine/

19 exp cigarette/

20 exp drinking behavior/

21 exp alcohol abuse/

22 exp alcoholism/

23 exp alcohol consumption/

24 exp wine/

25 exp exercise/

26 exp aerobic exercise/

27 exp sport/

28 exp fitness/

29 exp body mass/

30 exp obesity/

31 exp abdominal obesity/

32 exp childhood obesity/

33 exp adolescent obesity/

34 exp hypertension/

35 exp blood pressure/

36 exp diabetes mellitus/

37 exp insulin/

38 exp glucose blood level/

39 exp hemoglobin A1c/

40 exp lipid/

41 exp dyslipidemia/

42 exp hyperlipidemia/

43 exp cholesterol/

44 exp lipoprotein/

45 exp triacylglycerol/

46 exp low density lipoprotein/

47 exp high density lipoprotein/

48 exp very low density lipoprotein/

49 exp metabolic syndrome X/

50 exp diet/

51 exp nutrition/

52 exp malnutrition/

53 exp nutritional disorder/

54 exp dystrophy/

55 exp nutritional deficiency/

56 or/7-55

57 6 and 56

58 1 or 57

59 ((adult$1 or adulthood or middle aged or young adult$4 or older or old people or senior$1 or elderly) adj5 (chronic renal insufficiency or renal damage or kidney damage or kidney function or chronic kidney disease or CKD or chronic kidney failure or chronic kidney insufficiency or chronic kidney dysfunction or chronic renal failure or chronic renal dysfunction)).ab,kw,ti.

60 exp adult/

61 exp aged/

62 exp adulthood/

63 exp middle aged/

64 exp young adult/

65 or/60-64

66 exp kidney disease/

67 exp kidney failure/

68 exp chronic kidney failure/

69 exp proteinuria/

70 exp glomerulus filtration rate/

71 or/66-70

72 65 and 71

73 59 or 72

74 exp cohort analysis/

75 exp case control study/

76 exp longitudinal study/

77 exp follow up/

78 exp retrospective study/

79 longitudinal.ab,kw,ti.

80 cohort stud$3.ab,kw,ti.

81 cohort analy$3.ab,kw,ti.

82 (follow up$1 or followup$1).ab,kw,ti.

83 case control stud$3.ab,kw,ti.

84 retrospective.ab,kw,ti.

85 prospective.ab,kw,ti.

86 or/74-85

87 58 and 73 and 86

A search strategy for Web of Science

#1 TS=(adolescen*) OR TS=(p$ediatric or p$ediatrics) OR TS=(teenage*) OR TS=(child*) OR TS=(kid or kids) OR TS=(boy or boys) OR TS=(girl or girls) OR TS=(schoolchild*) OR TS=(schoolage*) OR TS=(school age*) OR TS=(youngster*) OR TS=(juvenile*)

#2 TS=(socioeconomic status or socio-economic status or SES) OR TS=(socioeconomic position* or socio-economic position* or SEP) OR TS=(family income) OR TS=(parental income) OR TS=(parental education) OR TS=(parental occupation) OR TS=(smok*) OR TS=(tobacco) OR TS=(cigarette or cigarettes) OR TS=(alcohol intake) OR TS=(alcohol drink*) OR TS=(drink* alcohol) OR TS=(alcohol consumption) OR TS=(wine or wines) OR TS=(liquor or liquors) OR TS=(spirit or spirits) OR TS=(physical activit* or PA) OR TS=(aerobic exercise*) OR TS=(aerobic sport*) OR TS=(body mass index or BMI) OR TS=(obesity) OR TS=(adiposity) OR TS=(high blood pressure) OR TS=(hypertension) OR TS=(diabetes or T2D or T1D) OR TS=(Glycated Hemoglobin A or Hemoglobin A1c or HbA1c) OR TS=(high blood glucose) OR TS=(low-density lipoprotein or LDL) OR TS=(high-density lipoprotein or HDL) OR TS=(very low density lipoprotein or VLDL) OR TS=(metabolic syndrome or MetS or Met or syndrome X) OR TS=(triglycerides or triacylglycerol or triacylglyceride or TAG or TG) OR TS=(diet*) OR TS=(nutrition or nutritions) OR TS=(nutritional deficienc*) OR TS=(dietary deficienc*) OR TS=(dystrophy)

#3 TS=(adult or adults) OR TS=(adulthood) OR TS=(middle aged) OR TS=(young adult*) OR TS=(older) OR TS=(old people) OR TS=(senior or seniors) OR TS=(elderly)

#4 TS=(chronic renal insufficiency) OR TS=(renal damage) OR TS=(middle aged) OR TS=(kidney damage) OR TS=(kidney function) OR TS=(chronic kidney disease or CKD) OR TS=(chronic kidney failure) OR TS=(chronic kidney insufficiency) OR TS=(chronic kidney dysfunction) OR TS=(chronic renal failure) OR TS=(chronic renal dysfunction)

#5 TS=(longitudinal) OR TS=(cohort stud*) OR TS=(follow up or follow ups) OR TS=(followup*) OR TS=(case control stud*) OR TS=(prospective) OR TS=(retrospective)

#6 #1 AND #2 AND #3 AND #4 AND #5

**Appendix 2. Inclusion/Exclusion form for primary studies**

Study ID:

Reviewer:

Date:

**Identification Details:**

Author:

Year:

Journal of Reference:

Source:

Digital Object Identifier (DOI):

On Endnote database: ………………………………………………..…………………………………………..…………….Yes/No

**Selection Criteria:**

1. Design is cohort study ……...…………………………………………………..Yes/No
2. The exposure is childhood modifiable risk factors (socioeconomic position, adiposity, smoking, alcohol consumption, nutrition, physical activity, fitness, blood pressure, diabetes, and dyslipidaemia) ………………………………………..………….Yes/No
3. The outcome is adulthood chronic kidney disease (CKD) or surrogate markers of CKD ……………………………….…………………………………………...Yes/No
4. Reporting required information …………..…………………………………….Yes/No

**Please tick only one box:**

| In | Out | Pending |
| --- | --- | --- |
|  |  |  |

**Appendix 3 NEWCASTLE-OTTAWA QUALITY ASSESSMENT SCALE COHORT STUDIES (Adapted)**

| **NEWCASTLE-OTTAWA QUALITY ASSESSMENT SCALE COHORT STUDIES (Adapted)** | | | | | |
| --- | --- | --- | --- | --- | --- |
|  |  | **Star awarded system** | **Star awarded** | | **Star** |
| **Selection** | | | | | |
| 1) Representativeness of the exposed cohort  (maximum one star) | a) truly representative of the average ________ (describe) in the community | * |  |  | |
|  | b) somewhat representative of the average ________ in the community | * |  |  |  |
|  | c) selected group of users e.g., nurses, volunteers | (no star) |  |  |  |
|  | d) no description of the derivation of the cohort | (no star) |  |  |  |
| 2) Selection of the non-exposed cohort (maximum one star) | a) drawn from the same community as the exposed cohort | * |  |  | |
|  | b) drawn from a different source | (no star) |  |  |  |
|  | c) no description of the derivation of the non-exposed cohort | (no star) |  |  |  |
| 3) Ascertainment of exposure (maximum one star) | a) secure record (e.g., surgical records) | * |  |  | |
|  | b) structured interview | * |  |  |  |
|  | c) written self-report | (no star) |  |  |  |
|  | d) no description | (no star) |  |  |  |
| 4) Demonstration that outcome of interest was not present at start of study (maximum one star) | a) yes | * |  |  | |
|  | b) no | (no star) |  |  | |
| **Comparability** | | | | | |
| 1) Comparability of cohorts on the basis of the design or analysis (maximum two star) | a) study controls for age and sex | * |  |  | |
|  | b) study considers other relevant covariates. | * |  |  | |
| **Outcome** | | | | | |
| 1) Assessment of outcome (maximum one star) | a) independent blind assessment | * |  |  | |
|  | b) record linkage | * |  |  | |
|  | c) self-report | (no star) |  |  | |
|  | d) no description | (no star) |  |  | |
| 2) Was follow-up long enough for outcomes to occur (maximum one star) | a) yes (select an adequate follow up period for outcome of interest) | * |  |  | |
|  | b) no | (no star) |  |  | |
| 3) Adequacy of follow up of cohorts (maximum one star) ^#^ | a) complete follow up – all subjects accounted for | * |  |  | |
|  | b) subjects lost to follow up unlikely to introduce bias – small number lost - > 80 % (select an adequate %) follow up, or description provided of those lost) | * |  |  | |
|  | c) follow up rate < 80 % (select an adequate %) and no description of those lost | (no star) |  |  | |
|  | d) no statement | (no star) |  |  | |
| **Final score:** | | | | | |

^#^ 0.5* is given if lost to follow-up > 20% and with description of those lost.
